# A comprehensive cognitive analysis of cervical dystonia: A single centre study

**DOI:** 10.1016/j.prdoa.2023.100226

**Published:** 2023-10-16

**Authors:** Shameer Rafee, Madeleine Diepman, Derval McCormack, Ruth Monaghan, Conor Fearon, Michael Hutchinson, Fiadhnait O'Keeffe

**Affiliations:** a Department of Neurology, St Vincent’s University Hospital, Ireland; b Department of Psychology, St Vincent’s University Hospital, Ireland; c School of Medicine and Medical Sciences, University College Dublin, Ireland

**Keywords:** Cervical dystonia, Cognition, Mood, Quality of life, Motor severity

## Abstract

**Introduction:**

Cervical dystonia (CD) presents as a motor disorder but has a number of non-motor features. Studies have demonstrated diverse changes in cognition in patients with CD. The rarity of this disorder, phenotypic heterogeneity, and, in particular, a lack of consistency in cognitive testing measures limits clear definition of cognitive changes in this disorder. The relationship between cognition, motor symptoms and quality of life has not been well defined. We undertook a comprehensive analysis of cognition in CD.

**Methods:**

Patients with adult onset idiopathic isolated CD (AOICD) who had completed a battery of cognitive assessments- general intellectual functioning, verbal and visual memory, executive functions and social cognition measures, were included. Participants were assessed for mood symptoms, motor severity and quality of life.

**Results:**

13 patients (8 women) with AOICD were included covering 40 cognitive subtests. Mean age was 59.9 years and mean TWSTRS-2 severity was 11. Mean estimated premorbid function was in the normal range. Overall performance on most measures were within normal limits. The lowest mean z-score was observed in Florida Affect Battery (social cognition) subtests, z = −1.75 and −0.81. and in verbal recall, z = −0.82. The majority of patients (75%) scored below population mean on spatial working memory and (62%) performed below population mean on word retrieval and working memory.

**Conclusion:**

We provide detailed cognitive results across a wide range of measures. Although patients tended towards average outcomes on the majority of tests, poorer performance than expected averages were noted in measures of social cognition, word retrieval, spatial working memory and, processing speed.

## 1 Introduction

People with adult onset isolated idiopathic cervical dystonia (AOICD), may have a number of sensory, psychiatric and cognitive, non-motor symptoms [1]. Studies examining cognitive changes in AOICD have shown deficits in executive function [2], aspects of social cognition [3]; and changes in information processing speed [4], [5] and memory [4] have also been noted. In AOICD, psychiatric symptoms such as depression and anxiety have a disproportionate influence on quality of life (QoL), more than dystonia motor severity [6].

Cognitive deficits can affect QoL in other movement disorders. In essential tremor it has been noted that cognitive difficulties were a greater predictor of functional disability than motor symptoms [7]. A direct influence of cognitive symptoms on motor disability has not been noted in AOICD; however, studies are limited by sample size and heterogeneity [8].

Studies of cognitive function in focal dystonia have been inconsistent due to variations in study methodology, use of heterogenous focal dystonia phenotypes and small study populations. A lack of agreed standardised measures, and heterogeneity within AOICD (tremor-/non-tremor-predominant, differing dystonia phenotypes), makes generalisation across this rare condition difficult [9].

Dysfunction of pallidal-striatal connectivity [10], frontal lobe and cerebellar resting state networks [10], demonstrated by functional MRI studies, may explain some mechanisms underlying postulated cognitive deficits in AOICD.

Clarifying patterns of cognitive dysfunction in AOICD opens new ways to improving QoL through targeted neuropsychological assessment and interventions and, wider multidisciplinary support. We present below a comprehensive cognitive profile of patients with cervical dystonia.

## 2 Patients & methods

Patients with AOICD, aged 18–70 years, who had completed comprehensive neuropsychological testing were included. All participants were receiving botulinum toxin (BoNT) therapy at 3 monthly intervals. Patients were excluded if they were using medication known to affect cognition (e.g. anti-cholinergic drugs and benzodiazepines). Patients with other neurological/ psychiatric disorders and significant medical conditions were also excluded.

## 3 Methods

All neuropsychological assessments were administered by two trained assistant neuropsychologists, supervised by the principal clinical neuropsychologist on the day of routine visits for BoNT injections to mitigate any potential impact of treatment on outcomes (week 0 of 3-monthly injection cycle). Tests were performed at two time points both at week 0 of treatment cycle. Participants were selected based on by convenience sampling and all were screened for anxiety and depression using psychometric questionnaires.

### 3.1 Disease severity and quality of life

Participants were assessed by a neurologist for dystonia motor severity using Toronto Western Spasmodic Torticollis Rating Scale 2- severity, (TWSTRS2s) [11]. Quality of life was assessed using the Cervical Dystonia Impact Profile-58 (CDIP58), a validated, disease-specific measure.

### 3.2 Neuropsychological assessment battery

A comprehensive battery of cognitive tests was included assessing the following domains- premorbid function, general intellectual functioning, language, information processing speed, attention/working memory, memory, executive function and social cognition. A description of each test is available in Table 1 (supplementary material). We selected these cognitive assessments to create a detailed cognitive profile and to have several measures assessing each cognitive domains.

### 3.3 Mood assessment

The Hospital Anxiety and Depression Scale (HADS-A/HADS-D) with cut off scores of ≥ 8 was used to indicate presence of clinically relevant mood symptoms [12]. This is a brief, well validated, self-completed measure.

Individual participant outcomes for each subtest (raw scores) are presented and results grouped into above average (Z ≥ 0.67), average (0.66 ≥ Z ≤ −0.66) and below average (Z ≤ −0.67) are also shown.

### 3.4 Statistical methods

Results from cognitive assessments with normative data were transformed to Z-scores and stratified into above average, average and below average. A Shaprio-Wilks test was performed to check normality. As most results were normally distributed, Pearson’s correlation coefficient was calculated for the relationship between cognitive scores, mood, disease severity and QoL.

Not all participants completed every sub-test, specific *n* values are shown in results tables. Ethics approval was granted by the local ethics committee and all participants provided informed consent prior to study inclusion.

## 4 Results

Thirteen patients (8 women) participated. Mean age was 59.9-years (±8.7). Mean TWSTRS2s was 11 (lower scores indicate less severe disease- TWSTR2s scores range from 0 to 24). Mean CDIP-58 total was 38 (lower values indicate better QoL). Three patients had depression scores above the clinically elevated cut-off and 6 patients were above the clinical cut-off anxiety scores. 11/13 participants had attained at least a level 3 education (at least secondary/high school).

A profile of the participants, mood scores, disease severity and CDIP58 scores is shown in Table 1.

**Table 1:** Participant characteristics. *HADS-A = Hospital anxiety and depression scale- anxiety, HADS-D = Hospital anxiety and depression scale- depression, TWSTRS2s = Toronto Western Spasmodic Torticollis Rating Scale 2- Severity, CDIP-58 = Cervical dystonia impact profile 58.*

**Participant**	**Age**	**HADS-A**	**HADS-D**	**TWSTRS2s**	**CDIP-58 total**
**1**	69	1	2	17	25
**2**	72	2	0	16	28
**3**	74	8	6	14	39
**4**	52	10	5	14	57
**5**	66	4	4	11	47
**6**	62	0	2	6	23
**7**	65	0	0	11	3
**8**	43	13	11	8	58
**9**	61	14	11	8	38
**10**	60	1	0	11	83
**11**	37	13	7	11	50
**12**	59	2	4	10	13
**13**	59	8	11	6	29

**Fig. 1:**
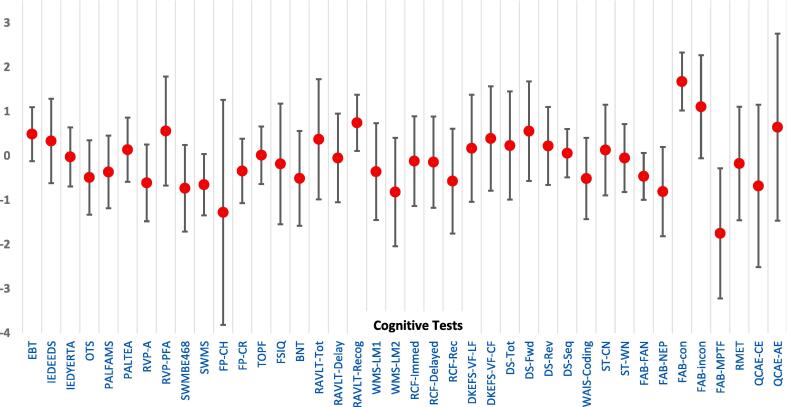
Mean and standard deviations in Z scores across all subtests. *BNT = Boston Naming Test, DKEFS-VF = Delis Kaplan Executive Function System- Verbal Fluency, EBT = emotional bias task, FP = Faux Pas, FAB = Florida Affect Battery, IED = intra-extra dimensional set shifting, OTS = one touch stockings, PAL = paired associative learning, QCAE = questionnaire of cognitive and affective empathy, RAVLT = Rey Auditory Verbal Learning Task, RCF = Rey Osterrieth Complex Figure, RMET = reading the mind in the eyes test, RVP = rapid visual processing, ST = stroop test, SWM = spatial working memory, TOPF = test of premorbid functioning, WAIS = Weschler Adult Intelligence Scale, WAIS-DS = Weschler Adult Intelligence Scale- Digit Span, WMS-LM1 = Weschler Memory Scale- Logical Memory 1, WMS-LM2 = Weschler Memory Scale = Logical memory 2.*

Raw mean scores and Z-scores based on mean scores showed variances in performance across a range of cognitive domains and subtests (Table 2 and Fig. 1). The lowest mean Z-scores were seen in social cognition measures (Florida Affect Battery (FAB)- matching emotional prosody to emotional face: z = -1.75, and FAB- naming emotional prosody: z = -0.81) and delayed verbal recall (WMS-LM2: z = -0.82). Mean estimated pre-morbid functioning showed performance within the normal range.

**Table 2:** Results of cognitive tests. Z scores were calculated from normal values provided by test licensers e.g. CANTAB; other Z scores were determined based on appropriate control values found in the literature (referenced in the table). r = ratio score/ probability score, p = percentile, a = actual score, s = scaled score; *BNT = Boston Naming Test, DKEFS-VF = Delis Kaplan Executive Function System- Verbal Fluency, EBT = emotional bias task, FP = Faux Pas, FAB = Florida Affect Battery, IED = intra-extra dimensional set shifting, OTS = one touch stockings, PAL = paired associative learning, QCAE = questionnaire of cognitive and affective empathy, RAVLT = Rey Auditory Verbal Learning Task, RCF = Rey Osterrieth Complex Figure, RMET = reading the mind in the eyes test, RVP = rapid visual processing, ST = Stroop test, SWM = spatial working memory, TOPF = test of premorbid functioning, WAIS = Weschler Adult Intelligence Scale, WAIS-DS = Weschler Adult Intelligence Scale- Digit Span, WMS = Weschler Memory Scale.*

**Cognitive domain** **Cognitive test**	**Subtest (n)**	**Mean score**	**Mean Z score ± SD**
**Estimated Premorbid Functioning**			
TOPF	TOPF (n = 13)	100.18^a^	0.01 ± 0.65
**Current general intellectual functioning**			
WASI- FSIQ II	FSIQ-II (n = 12)	97.25^a^	−0.18 ± 1.36
**Language**			
BNT	BNT (n = 13)	12.38^a^	−0.51 ± 1.07
**Information and processing speed**			
ST	Colour naming (n = 13)	10.38 ^s^	0.13 ± 1.02
	Word naming (n = 13)	9.85 ^s^	−0.05 ± 0.77
WAIS	Coding (n = 13)	8.46 ^s^	−0.51 ± 0.92
**Attention and working memory**			
WAIS- DS	Total (n = 13)	10.69 ^s^	0.23 ± 1.22
	Forward (n = 12)	11.67 ^s^	0.56 ± 2.64
	Reverse (n = 12)	10.67 ^s^	0.22 ± 0.88
	Sequence (n = 12)	10.17 ^s^	0.06 ± 0.55
CANTAB RVP	RVPA (n = 12)	0.86^a^	−0.61 ± 0.87
	RVPFFA (n = 12)	0.02^a^	0.56 ± 1.23
**Memory**			
RAVLT	RAVLT total (n = 13)	49.3^p^	0.37 ± 1.36
	RAVLT delayed recall (n = 13)	48.04^p^	−0.05 ± 0.99
	RAVLT recognition (n = 13)	73.98^p^	0.74 ± 0.63
WMS	LM1 (n = 13)	8.92 ^s^	−0.36 ± 1.09
	LM2 (n = 13)	7.54 ^s^	−0.82 ± 1.22
RCF	Immediate recall	45.38^p^	−0.12 ± 1.01
	Delayed recall	45^p^	−0.14 ± 1.03
	Recognition	36^p^	−0.57 ± 1.18
CANTAB PAL	PALFAMS (n = 12)	10.83^a^	−0.36 ± 0.82
	PALTEA28 (n = 10)	15.2^a^	0.14 ± 0.72
**Executive function**			
DKEFS-VF	Letter fluency (n = 12)	10.5 ^s^	0.17 ± 1.21
	Category fluency (n = 12)	11.17 ^s^	0.39 ± 1.18
CANTAB IED	IEDEEDS (n = 12)	9^a^	0.33 ± 0.95
	IEDYERTA (n = 12)	37^a^	−0.02 ± 0.67
CANTAB OTS	OTSPSFC (n = 12)	8.42^a^	−0.49 ± 0.84
CANTAB SWM	SWMBE468 (n = 12)	20.42^a^	−0.73 ± 0.98
	SWMS (n = 12)	9.33^a^	−0.65 ± 0.69
**Social cognition**			
FP	Correct Hits (n = 9)	0.8^r^	−1.28 ± 2.54^14^
	Correct Rejects (n = 9)	1.0^r^	−0.34 ± 0.73^14^
FAB	Facial naming affect	83.33^p^	−0.46 ± 0.53
	Naming emotional prosody (n = 9)	83.33^p^	−0.81 ± 1.01
	Conflicting emotional prosody (congruent) (n = 9)	94.4^p^	1.67 ± 0.65
	Conflicting emotional prosody (incongruent) (n = 9)	86.6^p^	1.1 ± 1.16
	Emotional prosody to emotional face (n = 9)	77.2^p^	−1.75 ± 1.5
RMET	RMET (n = 10)	68.42^a^	−0.17 ± 1.28
QCAE	Cognitive empathy (n = 10)	51.5^a^	-0.68^15^
	Affective empathy (n = 10)	37.3^a^	0.64^15^
CANTAB EBT	EBT (n = 12)	8.81^a^	0.49 ± 0.61

Results from individual patients demonstrated that the majority of participants scored poorly on a number of domains: word retrieval performance (BNT, 62% below average), spatial working memory (SWBE468, 75% below average) and processing speed (WAIS-coding, 62% below average). It should be noted that mean Z-score for BNT and WAIS-coding were within the normal range. The majority of participants performed better than the population mean in another test of processing speed (ST-C, 54% above average). Most participants also performed well in one subtest of memory (RAVLT-recognition, 64% above average). The performance ranges for every participant on all subtests is shown in Table 2 (supplementary data).

Social cognition results were particularly interesting: all 13 participants (100%) scored below average in FAB-emotional face to emotional prosody. The Faux Pas- correct hits test had the second lowest mean Z-score (Z = -1.28). However, participants performed better in other FAB subtests (conflicting emotional prosody congruent, 89% above average and, incongruent, 78% above average).

The cognitive measures were assessed for correlation with QoL and motor severity scores. After calculating Bonferroni corrected p value, no Pearson’s correlation *r* value met statistical significance.

## 5 Discussion

We present a detailed neuropsychological profile of patients with AOICD. We employed a range of assessments to provide a deep phenotyping of cognitive performance, highlighting difficulties, where present. The data can be interpreted in two, complementary, ways: overall mean Z-scores and individual performance. Using both, our study demonstrates deficits across a range of cognitive functions from aspects of social cognition (matching emotional prosody to emotional face and faux pas correct hits), processing speed, and spatial memory. Scores show that patients perform within the average range (on mean Z-scores) for all subtests within the following domains: premorbid functioning, general intellectual functioning, language, information and processing speed and, attention and working memory. In word retrieval, nearly two-thirds of participants were in the below average range, even though group mean Z-scores were within the average range.

Although no specific “cut-off” values exist for TWSTRS2s, our mean score (11) is lower than what has been described in a large registry, indicating milder disease [13]. Despite the mild motor severity, nearly half our cohort experienced clinically elevated anxiety or depressive symptoms or both- a common finding in cervical dystonia [14]. There is an association between anxiety/ depression and cognitive performance. Some dystonia publications have assessed cognition without also assessing impact of mood [15], [16], whereas studies assessing social cognition controlled for the impact for mood [17], [18]. A number of the participants our study had excess mood symptoms, and this may have impacted outcomes [19].

The QoL measure (CIDIP-58), another measure with no pre-defined thresholds, is lower in our patients than in other reports in the literature [23], [20], again possibly indicating a milder form of disease. In our correlation analysis, we noted no clear significant correlations between mood, cognitive and motor scores.

Neurologists treating AOICD patients typically do not view this population as one that might have significant cognitive dysfunction [1], but evidence has progressed significantly over the past two decades. Studies have consistently reported varied but persistent deficits [3], [4], [8], [15] A recent systematic review, of twenty studies assessing cognition, summarises the available evidence [9]. Many of these studies, however, exclude patients that show obvious cognitive deficits, introducing bias. Also, most tended to use brief cognitive screening assessments [9]. The AOICDs do not typically demonstrate obvious cognitive impairment; screening tools might are likely not sensitive enough to pick up any abnormalities.

The primary focal dystonias are considered a “network disorder” with functional changes in the striatum and ventral intraparietal area [21], structures which also mediate cognition [22], [23]. Deficits in social cognition have been previously noted in AOICD [5], [8] but as we have shown here, results are mixed. A recent study showed intact social cognitive abilities but found that facial expressions of fear were less recognised than other facial emotions [18]. Our study noted that patients performed better than normative data in some social cognition subtests and significantly worse in others.

AOICD is an inherently heterogenous disease with variations in motor and non-motor symptoms [24]. It is possible that different phenotypes differ in pathophysiology and these could affect outcomes. This may explain variations encountered in our study. The largest cognitive study in cervical dystonia contained 60 patients but accounting for the possibility of spread of dystonia and influence of medications, this number might be insufficient [2].

Another difficulty in the CD and cognition literature is the limited nature and variety in tests. Reconciling these can be difficult. There is debate on selecting appropriate testing (so called “gold standard”) measures in cognitive testing [25]. It has been suggested that neuropsychological assessments can be a barrier to study participation- duration of assessments is the main reason cited and may prove an obstacle to larger recruitment [26]. More women develop AOICD [27]. Women with mood symptoms may also develop AOICD earlier than men and women with anxiety/depression [19]. Our study had a female preponderance which may have influenced results, but this needs further research.

Our study overcomes one of these limitations by using 40 standardized and validated neuropsychological tools- providing one of the most comprehensive reviews to date. We also strived towards a homogenous population. Our main limitation is the small numbers and the lack of healthy control subjects. Many standardized psychometric measures e.g. Wechsler tests and CANTAB, provide detailed normative values that obviate the need for control participants, but this was not available for every subtest. For studies where normative data was not available (e.g. faux pas) we used appropriate control results from the literature, a common method in the cognitive assessment literature [28], [29].

## 6 Conclusion

We provide an extensive evaluation of cognition in this rare hyperkinetic disorder. From the lack of consistent findings this an area that warrants larger, detailed studies. Cognitive changes have the potential to provide insights into disease mechanism which has largely remained elusive. Given the potential for cognitive changes to impact quality of life, clinicians should consider these in their interactions with AOICD patients. A syndromic approach to AOICD is warranted, one which encompasses the motor and sensory features but also the psychiatric and cognitive aspects.

Design: RM, DM, FOK

Execution: SR, MD, RM, DM, FOK

Analysis: SR, MD, RM, DM

Writing: SR, MH, CF, FOK

Editing of final version: SR, CF, MH, FOK

SR has received an educational travel grant from Abbvie.

CF has received research grants from the Michael J Fox Foundation.

There are no other relevant financial disclosures.

Data may be provided on reasonable request, is subject to ethics committee approval and institutional data sharing agreement.

## Declaration of Competing Interest

The authors declare that they have no known competing financial interests or personal relationships that could have appeared to influence the work reported in this paper.

## References

[1] Stamelou M., Edwards M., Hallett M., Bhatia K. (2012). The non-motor syndrome of primary dystonia: Clinical and pathophysiological implications. Brain.

[2] Yang J., Shao N.a., Song W., Wei Q., Ou R., Wu Y., Shang H.-F. (2017). Nonmotor symptoms in primary adult-onset cervical dystonia and blepharospasm. Brain Behav..

[3] Burke T., Monaghan R., McCormack D., Cogley C., Pinto-Grau M., O'Connor S., Donohoe B., Murphy L., O'Riordan S., Ndukwe I., Hutchinson M., Pender N., O'Keeffe F. (2020). Social cognition in cervical dystonia: A case-control study. Clin. Park Relat. Disord..

[4] Monaghan R., Cogley C., Burke T., McCormack D., O'Riordan S., Ndukwe I., Hutchinson M., Pender N., O'Keeffe F. (2021). Non-motor features of cervical dystonia: Cognition, social cognition, psychological distress and quality of life. Clin. Park Relat. Disord..

[5] Czekóová K., Zemánková P., Shaw D.J., Bareš M. (2017). Social cognition and idiopathic isolated cervical dystonia. J. Neural Transm..

[6] Ndukwe I., O’Riordan S., Walsh C.B., Hutchinson M. (2020). Trust the Patient Not the Doctor: The Determinants of Quality of Life in Cervical Dystonia. Front. Neurol..

[7] Louis E.D. (2010). Functional correlates of lower cognitive test scores in essential tremor. Mov. Disord..

[8] Foley J.A., Saman Vinke R., Limousin P., Cipolotti L. (2017). Relationship of cognitive function to motor symptoms and mood disorders in patients with isolated dystonia. Cogn. Behav. Neurol..

[9] O’Connor S., Hevey D., Burke T., Rafee S., Pender N., O’Keeffe F. (2023). A Systematic Review of Cognition in Cervical Dystonia. *Neuropsychol. Rev*..

[10] Li Z., Prudente C.N., Stilla R., Sathian K., Jinnah H.A., Hu X. (2017). Alterations of resting-state fMRI measurements in individuals with cervical dystonia. Hum. Brain Mapp..

[11] Albanese A., Sorbo F.D., Comella C., Jinnah H.A., Mink J.W., Post B., Vidailhet M., Volkmann J., Warner T.T., Leentjens A.F.G., Martinez‐Martin P., Stebbins G.T., Goetz C.G., Schrag A. (2013). Dystonia rating scales: Critique and recommendations. Mov. Disord..

[12] Bjelland I., Dahl A.A., Haug T.T., Neckelmann D. (2002). The validity of the Hospital Anxiety and Depression Scale. J. Psychosom. Res..

[13] Agarwal P., Barbano R., Moore H., Schwartz M., Zuzek A., Sadeghi M., Patel A. (2022). OnabotulinumtoxinA Dosing, Disease Severity, and Treatment Benefit in Patients With Cervical Dystonia: A Cohort Analysis From CD PROBE. Front. Neurol..

[14] Berman B.D., Junker J., Shelton E., Sillau S.H., Jinnah H.A., Perlmutter J.S., Espay A.J., Jankovic J., Vidailhet M., Bonnet C., Ondo W., Malaty I.A., Rodríguez R., McDonald W.M., Marsh L., Zurowski M., Bäumer T., Brüggemann N. (2017). Psychiatric associations of adult-onset focal dystonia phenotypes. J. Neurol. Neurosurg. Psychiatry.

[15] Hinse P., Leplow B., Humbert T., Lamparter U., Junge A., Emskötter T. (1996). Impairment of visuospatial function in idiopathic spasmodic torticollis. J. Neurol..

[16] Maggi G., D'Iorio A., Mautone G., Peluso S., Manganelli F., Dubbioso R., Esposito M., Santangelo G. (2019). Cognitive correlates of prospective memory in dystonia. Park Relat Disord..

[17] Rinnerthaler M., Benecke C., Bartha L., Entner T., Poewe W., Mueller J. (2006). Facial recognition in primary focal dystonia. Mov. Disord..

[18] Ellement B., Jasaui Y., Kathol K., Nosratmirshekarlou E., Pringsheim T., Sarna J., Callahan B.L., Martino D. (2021). Social cognition in cervical dystonia: phenotype and relationship to anxiety and depression. Eur. J. Neurol..

[19] Ndukwe I., O’Riordan S., Walsh C., Hutchinson M. (2020). Mood disorder affects age at onset of adult-onset cervical dystonia. Clin Park Relat Disord..

[20] Simonetta-Moreau M., Picaut P., Volteau M., Poewe W. (2019). Quality of life improvements in patients with cervical dystonia following treatment with a liquid formulation of abobotulinumtoxinA (Dysport ®). Eur. J. Neurol..

[21] Norris S.A., Morris A.E., Campbell M.C., Karimi M., Adeyemo B., Paniello R.C., Snyder A.Z., Petersen S.E., Mink J.W., Perlmutter J.S. (2020). Regional, not global, functional connectivity contributes to isolated focal dystonia. Regional, Not Global, Functional Connectivity Contributes to Isolated Focal Dystonia..

[22] Provost J.S., Hanganu A., Monchi O. (2015). Neuroimaging studies of the striatum in cognition Part I: Healthy individuals. Front. Syst. Neurosci..

[23] Rizzolatti G., Fogassi L., Gallese V. (2002). Motor and cognitive functions of the ventral premotor cortex. Curr. Opin. Neurobiol..

[24] Groen J.L., Kallen M.C., van de Warrenburg B.P.C., Speelman J.D., van Hilten J.J., Aramideh M., Boon A.J.W., Klein C., Koelman J.H.T.M., Langeveld T.P., Baas F., Tijssen M.A.J. (2012). Phenotypes and genetic architecture of focal primary torsion dystonia. J. Neurol. Neurosurg. Psychiatry.

[25] Levin H.S. (1994). A Guide to Clinical Neuropsychological Testing. Arch. Neurol..

[26] Olson R., Parkinson M., McKenzie M. (2010). Selection bias introduced by neuropsychological assessments. Can. J. Neurol. Sci..

[27] Rafee S., O’Riordan S., Reilly R., Hutchinson M. (2021). We Must Talk about Sex and Focal Dystonia. Mov. Disord..

[28] Boringa J.B., Lazeron R.HC., Reuling I.EW., Adèr H.J., Pfennings L.E., Lindeboom J., de Sonneville L.MJ., Kalkers N.F., Polman C.H. (2001). The Brief Repeatable Battery of Neuropsychological Tests: normative values allow application in multiple sclerosis clinical practice. Mult. Scler. J..

[29] Wilk C.M., Gold J.M., Humber K., Dickerson F., Fenton W.S., Buchanan R.W. (2004). Brief cognitive assessment in schizophrenia: Normative data for the Repeatable Battery for the Assessment of Neuropsychological Status. Schizophr. Res..

