# Supplementary material for: A comprehensive cognitive analysis of cervical dystonia: A single centre study
Source: Clin Park Relat Disord. 2023 Oct 16;9:100226. doi: 10.1016/j.prdoa.2023.100226 (PMC10594573; doi:10.1016/j.prdoa.2023.100226)
Supplement: Supplementary data 1 [file mmc1.docx]

| **Estimated premorbid function** | Test of premorbid function (TOPF) |  |
| --- | --- | --- |
| **General Intellectual Functioning** | Weschler Abbreviated Scale of Intelligence full scale intelligence quotient-II (WASI FSIQ-II) | Assess general intellectual ability. |
| **Language** | Boston Naming Test (BNT)- 15 | Word retrieval performance. |
| **Information Processing Speed** | | |
|  | Weschler Adult Intelligence Scale (WAIS) coding | Measures processing speed |
|  | Stroop test-colour naming (ST-CN) | Processing speed: assesses the ability to inhibit cognitive interference that occurs when the processing of a specific stimulus feature impedes the simultaneous processing of a second stimulus attribute. |
|  | ST-word naming (ST-WN) | Processing speed: assesses the ability to inhibit cognitive interference that occurs when the processing of a specific stimulus feature impedes the simultaneous processing of a second stimulus attribute. |
| **Attention/ Working Memory** | | |
|  | WAIS- digit span total/ forward/ reverse/ sequencing | Measures working memory. |
| Cambridge Neuropsycholgoical Test Automated Battery (CANTAB) | Rapid visual information processing (RVP)   - RVPA - RVPFFA - RVPMDL | A measure of sustained attention.  Higher scores on RVPA indicate better performance.  Higher scores on RVPFFA indicate poorer performance.  Higher scores on RVPMDL indicate poorer performance. |
| **Memory** | | |
|  | Rey Auditory verbal learning task (RAVLT) | Measure of an individual’s ability to encode, combine, store and recover verbal information in different stages of immediate memory. Yields scores for *total* words recalled after interference, recall after 20 minute *delay*, and total words *recognised* from the list. |
|  | Weschler Memory Scale (WMS)- LM1 | Measure verbal episodic memory- immediate recall |
|  | WMS-LM2 | Measure verbal episodic memory- delayed recall. |
|  | Rey-Osterrieth Complex Figure | Measures visuospatial processing and visual memory- copy, immediate recall, delayed recall and recognition. |
|  | CANTAB Paired associates learning (PAL)   - PALTEA28 - PALFAMS | Assesses visual memory and new learning  Higher scores indicate poorer performance. |
| **Executive function** | | |
|  | Delis Kaplan Executive function system verbal fluency- letter fluency/ category fluency (DKEFS-VF- LF/CF) | Measure of verbal executive function. |
| CANTAB | Intra- extra dimensional set shift (IED)   - IEDEEDS - IEDYERTA | Test of rule acquisition and reversal. It features visual discrimination and attentional set formation maintenance, shifting and flexibility of attention. This test is sensitive to changes in the fronto-striatal areas of the brain and is a computerised analogue of the Wisconsin Card Sorting test.  Higher scores indicate poorer performance. |
| CANTAB | One touch stockings of Cambridge (OTS)   - OTSMDLFC - OTSPSFC | Test of executive function, based upon the Tower of Hanoi test. It assesses both the spatial planning and the working memory subdomains.  Higher scores indicate poorer performance on OTSMDLFC.  Higher score indicate better performance on OTSPSFC. |
| CANTAB | Spatial working memory (SWM)   - SWMBE468 - SWMS | Requires retention and manipulation of visuospatial information. This self-ordered test has notable executive function demands and provides a measure of strategy as well as working memory errors.  Higher scores indicate poorer performance on SWMBE468.  Higher scores indicate poorer performance on SWMS. |
| **Social cognition** | | |
|  | Faux pas (FP)   - Correct hits - Correct rejects | Evaluates the ability to infer the thoughts and feelings of others in a potentially sensitive social situation.  Higher scores indicate better performance. |
|  | Florida affect battery (FAB) | Assesses the perception of facial and prosodic affect under a variety of task demands.  Higher scores indicate better performance. |
|  | Reading the mind in the eyes test (RMET) | Assesses the ability to recognise complex mental states as expressed by human eyes.  Higher scores indicate better performance. |
|  | Questionnaire of cognitive and affective empathy (QCAE)   - Affective empathy - Cognitive empathy | Self-report measure of adults’ cognitive and affective empathy.  Higher scores indicate better performance. |
| CANTAB | Emotion bias task (EBT) | Detects perceptual bias in facial emotion perception.  Higher scores indicate a bias towards selecting “happy”. |

**Supplementary material Table 1.** All cognitive domains and subtests administered to participants.

| **Test** | **Above average** | **Average** | **Below average** |
| --- | --- | --- | --- |
| TOPF | 2/13 | 10/13 | 1/13 |
| WAIS- FSIQ II | 4/12 | 2/12 | 6/12 |
| BNT | 3/13 | 2/13 | 8/13 |
| ST- colour naming | 7/13 | 3/13 | 3/13 |
| ST- word naming | 3/13 | 7/13 | 3/13 |
| WAIS- coding | 1/13 | 4/13 | 8/13 |
| WAIS- DS | 5/13 | 5/13 | 3/13 |
| RVPA’ | 1/12 | 5/12 | 6/12 |
| RVPPFA | 3/12 | 8/12 | 1/12 |
| RAVLT Total | 5/13 | 4/13 | 4/13 |
| RAVLT Delay | 3/13 | 7/13 | 3/13 |
| RAVLT Recognition | 7/11 | 4/11 | 0/11 |
| WMS- LM1 | 3/13 | 5/13 | 5/13 |
| WMS- LM2 | 3/13 | 3/13 | 7/13 |
| RCF Immediate Recall | 1/13 | 7/13 | 3/13 |
| RCF Delayed recall | 1/13 | 10/13 | 2/13 |
| RCF Recognition | 2/13 | 5/13 | 6/13 |
| PALFAMS | 1/12 | 7/12 | 4/12 |
| PALTEA28 | 1/10 | 8/10 | 1/10 |
| DKEFS verbal fluency- LF | 5/12 | 4/12 | 3/12 |
| DKEFS verbal fluency- CF | 4/12 | 6/12 | 2/12 |
| IEDEEDS | 5/11 | 3/11 | 3/11 |
| IEDYERTA | 0/12 | 10/12 | 2/12 |
| OTSPSFC | 1/12 | 8/12 | 3/12 |
| SWMBE468 | 1/12 | 2/12 | 9/12 |
| SWMS | 1/12 | 5/12 | 6/12 |
| Faux pas Total | 0/9 | 5/9 | 4/9 |
| FAB- facial naming affect | 0/9 | 5/9 | 4/9 |
| FAB- naming emotional prosody | 0/9 | 5/9 | 4/9 |
| FAB- conflicting emotional prosody (congruent) | 8/9 | 1/9 | 0/9 |
| FAB- conflicting emotional prosody (incongruent) | 7/9 | 1/9 | 1/9 |
| FAB- emotional prosody to emotional face | 0/9 | 0/9 | 9/9 |
| RMET | 4/10 | 3/10 | 3/10 |
| QCAE cognitive empathy | 3/10 | 3/10 | 4/10 |
| QCAE affective empathy | 3/10 | 4/10 | 3/10 |
| Emotion bias task | 4/12 | 8/12 | 0/12 |

**Supplementary material Table 2.** Participants who scored above average (Z > 0.67), average (0.66 > Z < -0.66) and below average (Z < -0.67). supplementary data.
